# Alteration of primary afferent activity following inferior alveolar nerve transection in rats

**DOI:** 10.1186/1744-8069-6-9

**Published:** 2010-02-03

**Authors:** Kazuharu Nakagawa, Mamoru Takeda, Yoshiyuki Tsuboi, Masahiro Kondo, Junichi Kitagawa, Shigeji Matsumoto, Azusa Kobayashi, Barry J Sessle, Masamichi Shinoda, Koichi Iwata

**Affiliations:** 1 Department of Dysphagia Rehabilitation, Nihon University School of Dentistry, 1-8-13 Kanda-Surugadai, Chiyoda-ku, Tokyo, 101-8310, Japan; 2 Department of Physiology, School of Dentistry at Tokyo, Nippon Dental University, 1-9-20 Fujimi-cho, Chiyoda-ku, Tokyo, 102-8159, Japan; 3 Department of Physiology, Nihon University School of Dentistry, 1-8-13 Kanda-Surugadai, Chiyoda-ku, Tokyo, 101-8310, Japan; 4 Division of Functional Morphology, Dental Research Center, Nihon University School of Dentistry, 1-8-13 Kanda-Surugadai, Chiyoda-ku, Tokyo, 101-8310, Japan; 5 Division of Oral Physiology, Department of Oral Biological Science, Niigata University Graduate School of Medical and Dental Sciences, 2-5274 Gakkocho-dori, Chuo-ku, Niigata, 951-8514, Japan; 6 Department of Oral diagnosis, Nihon University School of Dentistry, 1-8-13 Kanda-Surugadai, Chiyoda-ku, Tokyo, 101-8310, Japan; 7 Department of Oral Physiology, Faculty of Dentistry, University of Toronto, 124 Edward Street, Toronto, Ontario, M5G 1G6, Canada; 8 Division of Applied System Neuroscience Advanced Medical Research Center, Nihon University Graduate School of Medical Science, 30-1 Ohyaguchi-Kamimachi, Itabashi-ku, Tokyo, 173-8610, Japan

## Abstract

**Background:**

In order to evaluate the neural mechanisms underlying the abnormal facial pain that may develop following regeneration of the injured inferior alveolar nerve (IAN), the properties of the IAN innervated in the mental region were analyzed.

**Results:**

Fluorogold (FG) injection into the mental region 14 days after IAN transection showed massive labeling of trigeminal ganglion (TG). The escape threshold to mechanical stimulation of the mental skin was significantly lower (i.e. mechanical allodynia) at 11-14 days after IAN transection than before surgery. The background activity, mechanically evoked responses and afterdischarges of IAN Aδ-fibers were significantly higher in IAN-transected rats than naive. The small/medium diameter TG neurons showed an increase in both tetrodotoxin (TTX)-resistant (TTX-R) and -sensitive (TTX-S) sodium currents (*I*_Na_) and decrease in total potassium current, transient current (*I*_A_) and sustained current (*I*_K_) in IAN-transected rats. The amplitude, overshoot amplitude and number of action potentials evoked by the depolarizing pulses after 1 μM TTX administration in TG neurons were significantly higher, whereas the threshold current to elicit spikes was smaller in IAN-transected rats than naive. Resting membrane potential was significantly smaller in IAN-transected rats than that of naive.

**Conclusions:**

These data suggest that the increase in both TTX-S *I*_Na _and TTX-R *I*_Na _and the decrease in *I*_A _and *I*_k _in small/medium TG neurons in IAN-transected rats are involved in the activation of spike generation, resulting in hyperexcitability of Aδ-IAN fibers innervating the mental region after IAN transection.

## Background

Numerous papers have described how peripheral nerve injury causes a variety of functional deficits in sensory processing [1-7]. Neuropathic pain also may occur after nerve injury [8-11], and whereas the injured tissue does usually repair, the neuropathic pain frequently persists [12-14]. One mechanism that is considered to underlie the abnormal pain after nerve damage involves regenerating nerve fibers. Injured nerves regenerate several weeks after nerve damage [15-17]. Some clinical reports have noted that areas innervated by the regenerated nerves show an altered sensitivity to a variety of stimuli compared to areas innervated by intact nerve fibers [18-21]. The regenerated fibers are morphologically similar to normal nerve fibers and terminals [22,23]. For example, periodontal sensory receptors are absent soon after inferior alveolar nerve (IAN) transection, but reappear more than 7 days after the transection, with morphological features similar to those in normal periodontal receptors [22]. However, varieties of functional changes are induced in injured nerves.

The background activity of injured primary afferent fibers [24] and their mechanical and heat-evoked responses are enhanced after nerve injury [25-27]. It is also reported that regenerated cutaneous afferent nerve fibers exhibit ectopic discharges in the sural nerve [28,29]. A variety of neuropeptides, such as neuropeptide Y or substance P, is also up- or down-regulated following peripheral nerve injury [30-32]. In the case of IAN transection for example, IAN fibers show significant increases in background activity and also trigeminal ganglion (TG) neurons show a change in the expression of several types of Na^+ ^channels [33-37]. These changes in peripheral nerves may account for changes not only in the excitability of the primary afferent neurons but also may contribute to excitability changes in the central nervous system (CNS) [36,38]. Hyperexcitability of peripheral nerves may be associated with sensitization of peripheral receptors [35], and CNS networks may become sensitized after long-lasting hyperexcitability of primary afferent neurons. Both peripheral and central sensitizations are thought to be involved in neuropathic pain following nerve injury [25,38-43].

The sodium (Na^+^) currents (*I*_Na_) have an important role in generating action potentials and are also involved in the modulation of primary afferent activity [44,45]. Therefore, *I*_Na _is thought to be important for modulating the excitability of primary afferent neurons after nerve injury [46-48]. The *I*_Na _is classified as either tetrodotoxin (TTX) -sensitive (TTX-S) or TTX-resistant (TTX-R) according to their sensitivity to TTX [49]. Many researchers have reported that TTX-R *I*_Na _as well as TTX-S *I*_Na _modulates primary afferent activity following nerve injury [46,48]. The potassium (K^+^) currents also are involved in modulation of the primary afferent neuronal excitability following nerve injury [24,50]. Following IAN injury, both the fast inactivating transient K^+ ^current (*I*_A_) and dominant sustained K^+ ^currents (*I*_K_) in TG neurons are decreased [24]. It is highly likely that *I*_Na_, *I*_A _and *I*_K _are up- and down-regulated in primary afferent neurons following IAN injury, resulting in hyperexcitability of the IAN fibers and induction of pain or other sensory abnormalities. However, the mechanisms underlying the functional changes in the transected IAN are not known.

Therefore, in the present study, the properties of the IAN innervating the mental region after IAN transection were analyzed using fluorogold (FG) tracing, nocifensive behavior monitoring, single fiber recording and patch-clamp recording from TG neurons in order to evaluate the changes in the excitability of TG neurons after IAN transection.

## Results

### FG labeling in TG neurons

**Figure 1:**
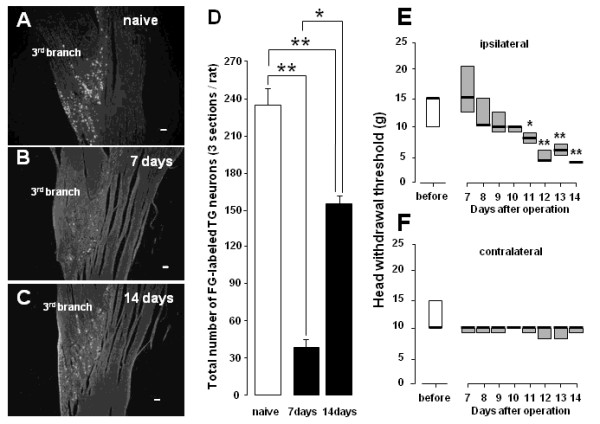
**Dark filed photomicrographs of FG-labeled TG neurons (A, B and C), the number of FG-labeled TG neurons in naive and IAN-transected rats (D) and change in the threshold intensity for eliciting escape behavior following mechanical stimulation to the mental skin reinnervated by the transected IAN (n = 5, E, F)**. The threshold intensity was plotted related to the stimulus intensity applied to rats face. A: naive rats, B: 7 days after IAN transection, C: 14 days after IAN transection. D: Total number of FG-labeled TG neurons in naive, 7 days and 14 days IAN-transected rats. * *p *< 0.05 (7 days vs. 14 days), ** *p *< 0.01 (vs. naive), E: ipsilateral side to IAN transection, F: contralateral side to IAN transection, before: before IAN transection. The escape threshold is shown as the medial value of the stimulus intensity. Burs in A, B and C indicate 100 μm. * *p *< 0.05, ** *p *< 0.01 (vs. before)

Many FG-labeled neurons were observed in the TG following FG injection into the mental skin in naive rats (Figure 1A). A small number of FG-labeled neurons were observed in the TG at 7 days after IAN transection and many were evident in the TG at 14 days after IAN transection (Figure 1B and 1C). Although the number of FG-labeled TG neurons at 7 and 14 days after IAN transection was significantly smaller than that in naive rats, the number of neurons at 14 days was significantly larger compared to that at 7 days (Figure 1D).

### Mechanical nocifensive behavior

After successful completion of the training, in which rats allowed noxious mechanical stimulation (> 15 g) to be applied to the mental skin region, the IAN was transected. Figure 1E (ipsilateral side to IAN transection) and F (contralateral side to IAN transection) illustrate the mechanical threshold intensity for evoking escape behavior by mechanical stimulation of the mental skin region before and 7-14 days after IAN transection. The threshold value was significantly lower at 11-14 days after IAN transection compared to pre-operative values (11 days: *p *< 0.05, 12-14 days: *p *< 0.01) (median values, pre-operative, ipsilateral: 15 g, contralateral: 10 g; 7 days after transection, ipsilateral: 15 g, contralateral: 10 g; 14 days after transection, ipsilateral: 4 g, contralateral: 10 g, n = 5 in each group).

### Primary afferent activity of the IAN

**Figure 2:**
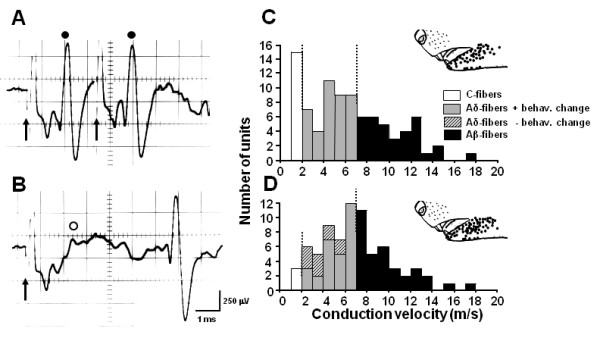
**Conduction velocity (CV) of A- and C-fiber units recorded from the IAN**. A: The antidromic spikes following 330 Hz electrical stimulation of the trigeminal spinal nucleus. Arrows indicate stimulus onset and closed circles represent antidromic spikes. B: The collision test for antidromic spikes. The open circle indicates the expected time point where antidromic spike should have appeared. C and D: Frequency histogram of CV in naive rats and IAN-transected rats, respectively. Inset figures in C and D indicate the receptive field in each unit.

**Figure 3:**
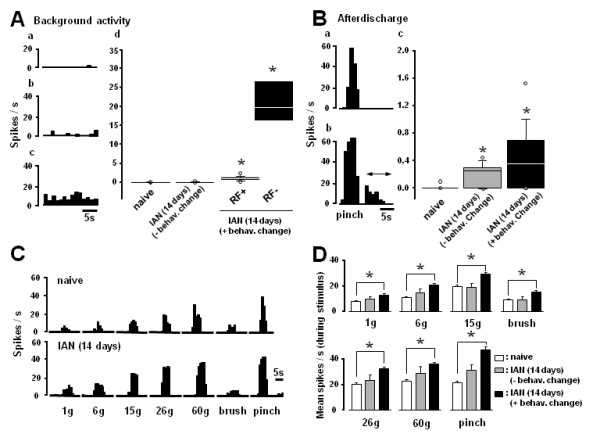
**Background activities (A) and afterdischarges (B) and mechanical responses (C and D) of Aδ-units to pressure, brushing or pinching of the receptive fields**. Aa and Ba: IAN fibers in naive rats, Ab and Bb: background activity (Ab) and afterdischarge (Bb) of IAN fibers with receptive fields at 14 days after IAN transection, Ac: background activity of the IAN fiber without receptive field at 14 days after IAN transection, Ad and Bc: mean background activities and afterdischarges in naive, IAN-transected rats without behavioral changes and IAN-transected rats, respectively. Note that background activities and afterdischarges of A-units in IAN-transected rats showed significantly higher firing frequency than those of naive rats. C: typical mechanical responses in naive and IAN-transected rats. D: mean mechanical responses of A-units in naive and IAN-transected rats. Note that A-units showed significantly higher responses to non-noxious and noxious mechanical stimulation and also IAN-transected rats without behavioral changes showed any changes in spike frequency. -behav.change: IAN-transected rats without behavioral changes after IAN transection, +behav.change: IAN-transected rats which showed mechano-allodynia like behavior after IAN transection. RF+: area of receptive field can be defined by mechanical simulation to face. RF-: area of receptive field cannot be defined by mechanical simulation to face. * *p *< 0.05 (vs. naive)

The activity of 76 single fiber activities was recorded from the IAN at 14 days after IAN transection (66 and 10 fibers from IAN-transected rats with and without behavioral changes, respectively) and the activity of 89 IAN fibers was recorded from naive rats. The single unit activities were classified as Aβ-, Aδ- and C-fiber responses according to their conduction velocities determined from the response latency and conduction distance (Figure 2). Fifteen fibers were classified as C-fibers and 34 fibers were as Aβ-fibers and 40 fibers were Aδ-fibers in naive rats (Figure 2C). On the other hand, only 3 fibers were classified as C-fibers and all others were classified as A-fibers (Aβ-: n = 34, Aδ-: n = 29) in IAN-transected rats (Figure 2D). We observed clear differences in background activity and mechanically evoked responses in Aδ-fibers between naive and IAN-transected rats. Five IAN fibers did not have any receptive field (RF) in the face and 24 fibers did have a facial RF. Most of the primary afferents of the IAN fibers did not show background firing in naive rats (Typical data shown in Figure 3Aa). In addition, we also did not observe background activities in IAN-transected rats without behavioral changes (- behav. change in Figure 3Ad). The background activities in the IAN-transected rats were significantly higher as compare with that in naive rats (Figure 3Ad) and those without a RF (RF-) showed the highest background activity (RF- in Figure 3Ac, d). The afterdischarge indicated by the arrow in Figure 3Bb was also significantly higher in the IAN-transected rats compared to naive rats (Figure 3Bc).

We analyzed the effect of mechanical stimulation of the RF on Aδ-fiber units only, because of the small population of C-fibers in IAN-transected rats. Aδ-fibers showed graded firing following increases in mechanical stimulus intensity from the non-noxious to the noxious range in naive and IAN-transected rats, as illustrated in Figure 3C. These fibers showed significantly larger responses to both non-noxious and noxious mechanical stimulation as compared to those of naive rats (Figure 3D).

### Patch-clamp recording from TG neurons

Mean size of FG-labeled TG neurons for patch-clamp recording was 27.4 ± 0.8 μm in naive rats and 27.8 ± 0.7 μm in IAN-transected rats (n = 17 each). Since there is a positive correlation between neuronal cell size and conduction velocity of A- and C- afferents in DRG neurons [51], small-diameter TG neurons recorded in the present study were considered to be classified as small to medium Aδ-TG neurons (diameter 21-36 μm) for the patch-clamp recording experiment. Following perforation of the cell membrane with amphotericin B, the series resistance dropped to < 20 MΩ (naive: 17.2 ± 0.8 MΩ; IAN transection: 16.8 ± 0.8 MΩ, n = 17 each) within 5-12 min and remained stable for more than 15 min. In addition, the value for the cell capacitance was 23.1 ± 1.0 pF in naive rats and 23.1 ± 1.3 pF in IAN-transected rats (n = 17 in each group).

### Change in I_Na_, I_A _and I_k_, and ability to generate action potentials in TG neurons

**Figure 4:**
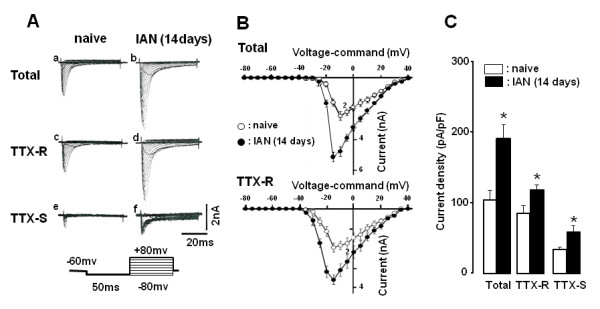
**Classification of TTX-S *I*_Na _and TTX-R *I*_Na _in naive and IAN-transected rats**. A: TTX-S *I*_Na _was isolated by digitally subtracting TTX-R *I*_Na _(in 1 μM TTX) from the total *I*_Na _(without TTX). B: The current-voltage (*I*-*V*) relationship of total *I*_Na _(without TTX) and TTX-R *I*_Na _(in 1 μM TTX) in both naive and IAN-transected rats. Mean values (mean ± SEM) of total and TTX-R *I*_Na _in TG neurons were illustrated in B. C: Peak current densities of total *I*_Na_, TTX-R *I*_Na _and TTX-S *I*_Na _in naive and IAN-transected rats. Open column: naive rats. Solid column: rats with IAN transection. * *p *< 0.05 (vs. naive)

Total *I*_Na _in TG neurons was larger in IAN-transected rats compared with naive rats, as illustrated in Figure 4Aa (naive rats) and Figure 4Ab (IAN-transected rats). The neurons were first held at -60 mV and then stepped from -80 mV to +80 mV for 50 ms (conditioning pre-pulse potential) as illustrated in Figure 4A. Since *I*_Na _can be subdivided into TTX-R *I*_Na _and TTX-S *I*_Na _on the basis of their sensitivity to TTX, we also analyzed these two currents in naive and IAN-transected rats. Representative wave forms of the total Na^+ ^currents, TTX-R *I*_Na _and TTX-S *I*_Na_, in TG neurons from naive and IAN-transected rats are illustrated in Figure 4A. TTX-R *I*_Na _recorded from TG neurons from IAN-transected rats was significantly larger than these recorded from naive rats (n = 7, *p *< 0.05) (Figure 4Ad). TTX-S *I*_Na _was also larger than that of naive rats (Figure 4Ae: naive, Figure 4Af: IAN-transected). TTX-R *I*_Na _was much larger than TTX-S *I*_Na _in both naive and IAN-transected rats (Figure 4Ac, d, e, f). The *I*-*V *relation curve and total *I*_Na _and TTX-R *I*_Na _are illustrated in Figure 4B. The total *I*_Na _(n = 7), TTX-R *I*_Na _(n = 7) and TTX-S *I*_Na _(n = 7) were all significantly larger in IAN-transected rats than naive rats (*p *< 0.05). The mean peak current densities of total *I*_Na _(n = 7), TTX-R *I*_Na _(n = 7) and TTX-S *I*_Na _(n = 7) were significantly larger in IAN-transected rats compared to naive rats as illustrated in Figure 4C (*p *< 0.05). Moreover, the magnitude of the transection-induced potentiation of mean peak current densities was significantly larger for TTX-S *I*_Na _than TTX-R *I*_Na _(78.9 ± 6.2% vs. 37.5 ± 7.5%, n = 7 in each, *p *< 0.05).

**Figure 5:**
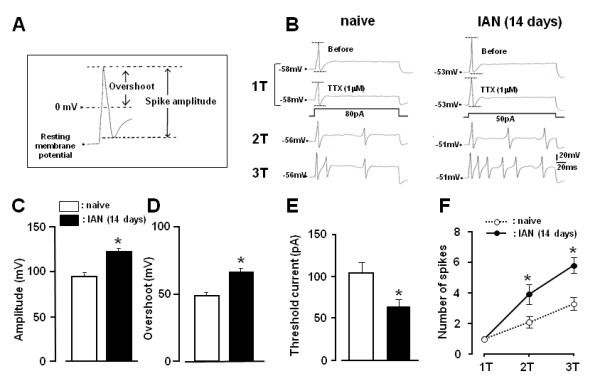
**Changes in spike form and the action potential firing in the TG neurons during application of depolarizing pulses in naive and IAN-transected rats under current clamp conditions**. The stimulus currents were applied at 50 pA steps under current clamp conditions. A and B: Sample recordings of action potentials of TG neurons from naive and IAN-transected rats. Spike amplitudes of action potentials were calculated from the distance between two dotted lines in B. The action potential was induced at the threshold (1T), two-times (2T) and three-times (3T) the threshold level. C: Mean spike amplitude of TG neurons from naive rats and IAN-transected rats. D: Mean overshoot of TG neurons from naive rats and IAN-transected rats. E: Mean threshold current in naive and IAN-transected rats. F: Mean number of spikes evoked in naive and IAN-transected rats during depolarization step pulses at 1T, 2T and 3T. * *p *< 0.05 (vs. naive)

Under current-clamp conditions, the ability of TG neurons to generate action potentials was analyzed. Action potentials were elicited during current injection into TG neurons in both naive and IAN-transected rats (Figure 5A). Before TTX administration, a single spike was generated at threshold current injection in naive and IAN-transected rats (upper traces in Figure 5B). The change in wave form was recorded during current injection of square pulses in 10 pA steps. The first spike amplitude at 1T stimulus and number of spikes at 2-3T stimuli were different between naive and IAN-transected rats (Figure 5B). All tested neurons exhibited action potentials in the presence of 1 μM TTX. In this case (upper 4 traces in Figure 5B), the spike amplitude was slightly decreased in naive and IAN-transected rats after administration of 1 μM TTX compared to those before TTX administration, suggesting that some neurons in both groups included TTX-S as well as TTX-R *I*_Na _components. The administration of 1 μM TTX inhibited 31 ± 6% of the control spike amplitude of action potentials at one threshold in naive rats. Furthermore, 1 μM TTX administration inhibited 23 ± 5% of the control spike amplitude of action potentials at one threshold in IAN-transected rats. We also analyzed the spike amplitude of action potentials, overshoot amplitude of action potentials, threshold intensity for spike generation and also number of spikes during gradual increases in membrane potential under the condition of the presence of 1 μM TTX (Figure 5C, D, E and 5F). Amplitudes of action potentials generated by 1T current application in the presence of 1 μM TTX were significantly larger in TG neurons in IAN-transected rats than in those of naive rats, as illustrated in Figure 5C (n = 10, *p *< 0.05). The overshoot amplitude of action potentials was also significantly larger in IAN-transected rats than that of naive rats, as shown in Figure 5D (n = 10, *p *< 0.05). The first spikes were elicited at significantly lower stimulus intensity in IAN-transected rats than in naive rats, as illustrated in Figure 5E (n = 7 in each group, *p *< 0.05). The resting membrane potential was significantly larger in TG neurons of IAN-transected rats compared to that in naive rats (IAN rats: -47.1 ± 1.1 mV, naive rats: -51.9 ± 1.3 mV, *p *< 0.05, n = 7 in each). Spike number was increased following an increase in the injected current in naive and IAN-transected rats. The mean number of spikes was significantly higher in IAN-transected rats than that of naive rats, as illustrated in Figure 5F (n = 10 in each threshold group, *p *< 0.05).

**Figure 6:**
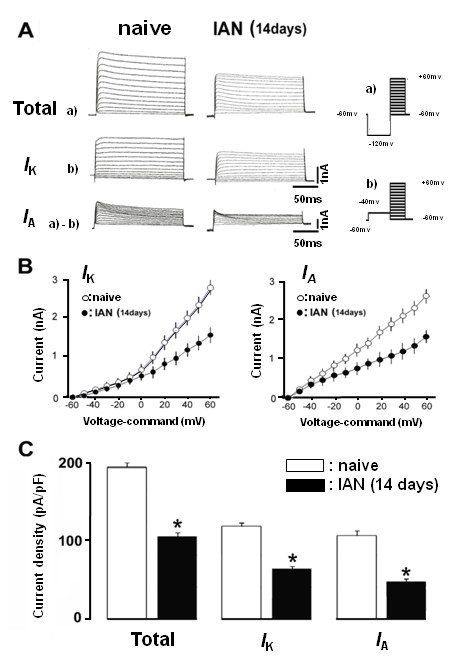
**The changes in voltage-gated K^+ ^currents of TG neurons with IAN-transected and naive rats**. Separation of total outward currents (a) into *I*_A_(a-b) and *I*_K _(b). (a) Initiated via a prepulse of -120 mV. (b) Initiated via a prepulse of -40 mV to +60 mV. Subtract a-b to reveal *I*_A_. Subtraction of Ab from Aa reveals a transient K^+ ^current (*I*_A_). B: Current-voltage relationships on *I*_K _and *I*_A _in TG neurons from naive (open circles) and IAN-transected rats (solid circles). Each value represents the mean ± SEM. C: Peak current densities for total K^+^, *I*_K _and *I*_A _in TG neurons from naive (open column) and the rats with IAN transection (solid column). Each value represents the mean ± SEM.* *p *< 0.05.

The changes in total K^+ ^current, *I*_k _and *I*_A _currents in TG neurons were also studied in IAN-transected rats (Figure 6). The peak values of total K^+ ^current, *I*_k _and *I*_A _currents were measured and all currents were significantly smaller in IAN-transected rats compared to naive rats, as illustrated in Figure 6A and 6B (mean ± SEM, total K^+ ^current: 4568 ± 188 pA in naive rats, 2522 ± 198 pA in IAN-transected rats, *p *< 0.05, n = 5; *I*_k_: 2902 ± 114 pA in naive rats, 1650 ± 127 pA in IAN-transected rats, *p *< 0.05, n = 5; *I*_A_: 2676 ± 155 pA in naive rats, 1012 ± 132 pA in IAN-transected rats, *p *< 0.05, n = 5). The mean peak current densities of total K^+ ^current (n = 5), *I*_k _and *I*_A _currents (n = 5) were significantly smaller in IAN-transected rats compared to naive rats, as illustrated in Figure 6C (*p *< 0.05).

## Discussion

The present results can be summarized as follows: 1) Injection of FG into the mental region 2 weeks after IAN transection produced a notable increase in retrograde labeling of TG neurons. 2) The escape threshold to mechanical stimulation of the mental skin was significantly lower at 11-14 days after IAN transection. 3) The background activity and afterdischarge of IAN Aδ-fibers were significantly higher in IAN-transected rats than naive rats. 4) Mechanical evoked responses of these fibers were also significantly larger in IAN-transected rats compared to those of naive rats. 5) The spike amplitudes in small/medium diameter TG neurons were significantly larger in IAN-transected rats compared to those of naive rats. 6) Voltage clamp recordings from TG neurons after IAN transection demonstrated an increase in both TTX-R and TTX-S Na^+ ^currents and a decrease in total K^+ ^current, *I*_K _and *I*_A_. 7) The threshold current to elicit spikes in TG neurons was significantly smaller in IAN-transected rats than that of naive rats. 8) Current injection into TG neurons induced high frequency spike discharges in rats with IAN transection. 9) Resting membrane potential was significantly smaller in IAN-transected rats than that of naive rats.

It has been reported that the injured primary afferent nerve fibers regenerate 2-3 weeks after injury [15-17]. We observed that more than half of TG neurons were labeled with FG at 14 days after IAN transection, the number of labeled neurons was also significantly increased compared with that at earlier days after injury, suggesting that many transected IAN fibers were reinnervating the mental region at this time period. It is also possible that the transected IAN may reinnervate other intraoral structures as well as the mental skin. It has been reported for example that the jaw-opening reflex elicited by tooth pulp stimulation can be recorded at 6 weeks after IAN transection in cats and that more than half of the IAN-transected cats show lingual nerve innervations into the tooth pulp [52]. However, in the present study we only analyzed TG neurons receiving input from the mental skin at 14 days after IAN transection, although we cannot rule out that some regenerated TG neurons become associated with inputs from intraoral or other facial structures as well as from mental skin. Furthermore, some previous studies have also reported that reinnervated nerve fibers change their response properties to a variety of peripheral stimuli such as mechanical and thermal stimuli [53-55]. It may be possible that the reinnervated IAN causes functional changes resulting in altered sensitivity to mechanical stimulation of the skin.

In the present study, there was a decrease in the escape threshold to mechanical stimulation of the mental skin following the IAN injury. Some previous clinical studies have reported that patients feel abnormal pain sensation after wound healing [18-20]. When considering the previous data and our findings, it is possible that an abnormal pain sensation occurs in the cutaneous tissues reinnervated by the injured nerves, but how the reinnervated nerves are involved in generation of an abnormal pain in the areas innervated by the injured IAN is not well understood. Therefore, we focused on the peripheral mechanisms underlying abnormal pain sensations in the mental region innervated following the IAN injury.

Our previous studies showed that A-fibers are predominantly involved in the hypersensitivity of the cutaneous RF innervated by the injured nerve as well as surrounding skin areas innervated by the uninjured nerves located close to the injured nerve [24,50]. In the present study, we observed that Aδ-fibers showed significantly increased background activity, afterdischarges following noxious stimulation and mechanical-evoked responses (see in Figure 3). Furthermore, TG neurons lacking RFs showed very high background activity in IAN-transected rats (Figure 3A). The precise mechanisms underlying these observations are unclear but one possible process could involve increases in interleukin-6 (IL-6) and nerve growth factor (NGF). It has been shown that such increases may be related to the development of mechanical allodynia after trigeminal nerve injury [56] and that Schwann cells at the nerve injury site release chemical signals including IL-6 or NGF which are retrogradely transported to the primary sensory neurons [57]. The possibility that the release of both IL-6 and NGF may be involved in the observed generation of ectopic discharges from injured IAN fibers lacking a RF is also supported by our findings that approximately a half of the TG neurons were unlabeled with FG at 14 days after IAN transection (Figure 1C). We have also reported recently that trigeminal spinal subnucleus caudalis (Vc) neurons display enhanced RF and response properties following reinnervation of their RFs by the transected IAN [58]. Thus, it is very likely that the increase in excitability of TG neurons after IAN transection as documented in the present study makes an important contribution to the increase in the Vc neuronal excitability. Furthermore, we observed that the number of C-fiber responses was decreased in IAN-transected rats compared to naive rats, as illustrated in Figure 2C and 2D. Saito et al. have also reported that heat responsive units in the Vc are significantly decreased in the IAN-regenerated rats, suggesting that the IAN C-fibers may have less ability to regenerate after transection reflecting the decrease in the number of C-fiber responses after IAN regeneration [58].

Alterations in ion channels may also be associated with nerve injury. It has been reported that many different types of ion channels are expressed in dorsal root ganglion (DRG) neurons after peripheral nerve injury [59,60] and TG neurons [35]. Na^+ ^and/or K^+ ^channels are importantly involved in spike generation and also in the modulation of neuronal excitability following nerve damage [59,60]. Chronic pain conditions are associated with altered Na^+ ^channel activity and the change in the Na^+ ^channel properties appears to be a critical feature of persistent pain following peripheral nerve injury [46-48].

It has been demonstrated that both TTX-S and TTX-R Na^+ ^currents were increased in small- and medium-diameter DRG neurons 2-7 weeks after sciatic nerve transection [46]. In agreement with this finding, the present study revealed that both TTX-R *I*_Na _and -S *I*_Na _densities of TG neurons were significantly larger in IAN-transected rats (14 days after transection) compared with those in naive rats (Figure 4). Our findings are consistent with the evidence that spike amplitudes of TG neurons were also significantly larger in IAN-transected rats following current injection. The threshold current for spike generation was significantly smaller in IAN-transected rats than that of naive rats and current injection into TG neurons induced high-frequency spike discharges in rats with IAN transection. Although the increase in the magnitude of TTX-S *I*_Na _was larger than that of TTX-R *I*_Na _in the TG neurons after IAN transection, it is possible that the hyperexcitability of TG neurons innervated by the regenerated IAN is augmented by an increase in TTX-R Na^+ ^current densities, resulting in abnormal excitation of the CNS networks and nociceptive behavior. However, further studies are needed to address this possibility.

In order to evaluate the changes in neuronal excitability associated with the demonstrated changes in sodium and potassium currents following IAN transection, we analyzed action potentials properties under current-clamp conditions. In this study, action potentials were generated by supra-threshold current injection. Current injection into TG neurons induced action potentials with larger amplitude in IAN-transected rats compared with those of naive rats. The current injection into TG neurons also induced high-frequency spike discharges and the threshold intensity to generate action potentials was significantly lower in IAN-transected rats. These changes in the physiological properties of TG neurons indicate that the excitability of TG neurons is increased in IAN-transected rats. There are several possible mechanisms that could explain the increase in action potential amplitude and frequency. One is an increase in the density of Na^+ ^channels and the other is a change in channel kinetics and permeability.

Voltage-gated K^+ ^channels are important physiological regulators of membrane potential in excitable tissue, including sensory ganglia [61,62]. TG neurons express two distinct classes of K^+ ^currents at varying levels, involving the *I*_K _and *I*_A _currents [63,64]. In this study, we found that the IAN-transection significantly decrease in the density of both *I*_K _and *I*_A _in the FG-labeled TG neurons. In agreement with this finding, we found that the resting membrane potential was significantly decreased following IAN-transection. In addition to increase in *I*_Na_, we can raise the possibility that the reduction of both *I*_K _and *I*_A _contributes to the hyperexcitability of IAN-transected TG neurons. The change in the excitability of TG neurons associated with the change in the *I*_Na _and K^+ ^current (*I*_K _and *I*_A_) may be involved in central sensitization of the Vc neurons that results in pain abnormalities following IAN transection.

## Conclusions

The present findings suggest that TTX-R *I*_Na _and -S *I*_Na_, and *I*_K _and *I*_A _in the reinnervated Aδ-IAN-TG neurons are involved in an increase in spike generation, resulting in the hyperexcitability of the reinnervated Aδ-IAN fibers. The findings further suggest that this hyperexcitability of the reinnervated Aδ-IAN fibers is involved in a development of mechano-allodynia in the area IAN are reinnervated following IAN transection.

## Methods

**Figure 7:**
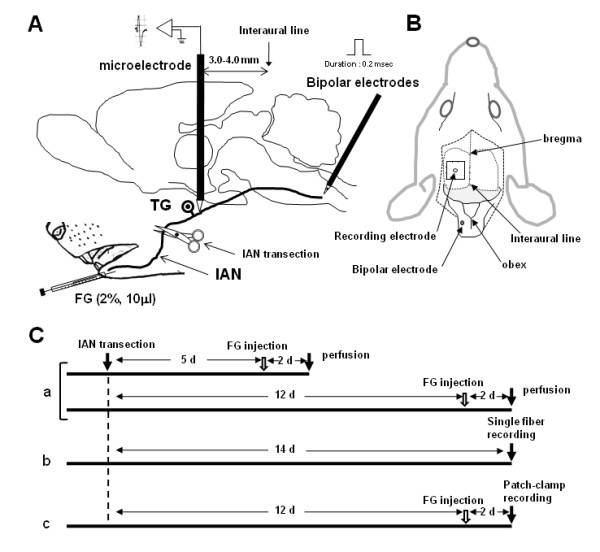
**The experimental diagram and time-course of the present study**. A: The schematic illustration of the FG injection site, electrode placement, B: Top view of the experimental set-up, C: The time-course of the present experiment. Ca: FG tracing experiment, Cb: single fiber recording experiment, Cc: patch-clamp recording experiment.

The detail experimental diagram and the time-course of the preset study were illustrated in Figure 7. This study was approved by the Animal Experimentation Committee at Nihon University School of Dentistry, and the treatment of the animals conformed to the guidelines of International Association for the Study of Pain [65].

### Inferior alveolar nerve transection

A total of 94 male Sprague-Dawley rats weighing 100-250 g was used for the present study. Rats were anesthetized with sodium pentobarbital (50 mg/kg, i.p.) and placed on a warm mat. A small incision was made on the surface of facial skin over the masseter muscle and the alveolar bone was reached through the masseter muscle. The surface of the alveolar bone was exposed and the bone covering the IAN was removed and the IAN was exposed. The IAN was transected at 7 mm proximal from the angle of alveolar bone and then immediately replaced into the inferior alveolar canal [58]. For patch clamp recording, FG dye (2%, 10 μl; Fluorochrome, Englewood, CO, USA) was subcutaneously injected into the mental region 2 days before the recording experiment. After surgery, Penicillin G potassium (20,000 units, i.m.; Eli Lilly, Indianapolis, IN) was injected to prevent infection.

### FG tracing

Fifteen rats were used for the FG tracing study (naive: n = 5, 7 days after transection: n = 5, 14 days after transection: n = 5). Rats were anesthetized with sodium pentobarbital (50 mg/kg, i.p.) and 10 μl of 2% FG was subcutaneously injected into the mental skin 2 days before perfusion in IAN-transected rats. Two days after the FG injection, rats were deeply anesthetized with the same anesthetic and perfused with 200 ml 0.9% saline followed by 500 ml of 4% paraformaldehyde. The TG was removed and post-fixed in the same fixative for 2 days and the tissue was then transferred to 20% sucrose (w/v) in phosphate-buffered saline (PBS) for several days for cryoprotection. Thirty-micron-thick sections were cut with a freezing microtome and sections were collected in PBS. FG immunohistochemical staining of TG neurons was carried out as previously described by Saito et al. [58]. TG neurons were defined as FG positive if the cytoplasm was stained with a black deposit. The FG-labeled TG neurons with clear nuclei were counted and their areas were measured. The number of FG-labeled TG neurons at the root of the third branch of the trigeminal nerve was counted in 3 sections (one section with the largest number of FG labeled neurons and next two sections) from each rat.

### Behavioral testing

In daily sessions, rats were trained to stay in a plastic cage and to keep their snout protruding through a hole on the wall during mechanical stimulation of the mental skin with von Frey filaments (Touch-Test, North Coast Medical, Inc., CA, USA). Rats under this condition can escape from von Frey stimulus freely. When rats escaped from the von Frey stimulus, the escape behavior was defined as nocifensive. The maximum intensity used before IAN transection was 15 g. The escape threshold was measured before the IAN was transected and then the IAN of those rats was transected. The escape threshold to mechanical stimulation of the mental skin was measured daily at 8 days before and 7-14 days after IAN transection. Only rats with IAN transection which showed a significant decrement in mechanical escape threshold at 14 days after IAN transection were used for single fiber and patch clamp recording experiments (see below). Quantitative mechanical stimuli were applied to the mental skin region in ascending and descending orders to evaluate the escape threshold. Each von Frey filament was applied 5 times. When rats showed an escape response to a filament, the bending force of that filament was defined as the escape threshold intensity [58]. The median threshold intensity was calculated from the values following one ascending and one descending trial.

### Single fiber recording after IAN transection

We used IAN-transected rats which showed hypersensitivity to mechanical stimulation of the mental skin and IAN-transected rats without behavior changes which did not show any behavioral changes after IAN transection for single fiber recording experiments. Rats were divided into 3 groups: ipsilateral to IAN transection (n = 16), ipsilateral to IAN without behavioral changes after IAN transection (n = 5) and naive groups (n = 22). For IAN fiber recording, each group of rats was anesthetized with sodium pentobarbital (50 mg/kg, i.p.) and the trachea and left femoral veins were cannulated to allow artificial respiration and intravenous administration of drugs, respectively. Anesthesia was maintained with halothane (2-3%) mixed with oxygen during surgery. Rats were mounted in a stereotaxic frame and a craniotomy was performed, 1-4 mm lateral to the midline and 2-5 mm anterior to the interaural line. The skull was rigidly secured to a head holder by stainless-steel screws and dental acrylic resin, and the nose holder was removed. This setup allowed convenient access to fibers which responded to stimulation of the orofacial RFs innervated by the IAN.

After surgery, anesthesia was maintained throughout the experiment by continuous inhalation of halothane (1-2%) mixed with oxygen. During recording sessions, rats were immobilized with pancuronium bromide (1 mg/kg/h, i.v.) and ventilated artificially. Expired CO_2 _concentration was monitored (Capstar-100, Cwe, Bioseb, USA) and maintained between 3.0-4.0%. Rectal temperature was maintained at 37-38°C by a thermostatically-controlled heating pad (ATB-1100, Nihon Kohden, Tokyo, Japan) and an electrocardiogram was monitored.

Bipolar electrodes (interpolar distance: 0.5 mm) were inserted into the spinal trigeminal subnucleus caudalis (Vc) 0.8 mm deep from the brainstem surface at the obex level to elicit antidromic spikes from Vc (Figure 7A). An enamel-coated tungsten microelectrode (impedance = 10 MΩ, 1000 Hz) was then advanced carefully through the cortex about 2.5-3.0 mm lateral to the midline and 3.0-4.0 mm anterior to the interaural line until an electrode tip reached the IAN trunk. Then, the electrode was advanced at 1 μm steps and single fiber activity was recorded. IAN unit activities were searched for by applying mechanical stimulation (pressure or brush) to the mental region. When single unit activity was isolated, responses to mechanical stimulation of the facial skin were carefully examined. Because of technical difficulties to approach intraoral structures, only cutaneous facial RFs were mapped. To identify antidromic responses, 1 ms electrical pulses (0.1-0.5 mA) were applied to the Vc. Each neuron was classified as Aβ- (> 7 m/s), Aδ- (7-2 m/s) or C- (< 2 m/s) fibers according to the conduction velocity of the action potentials calculated from the antidromic latency and the distance between recording and stimulating sites [66].

Graded mechanical stimuli were applied to the most sensitive areas of RFs. Mechanical stimuli consisted of quantitative pressure with von Frey filaments (1, 6, 15, 26 and 60 g), brushing with a camel hair brush and pinch produced by a small arterial clip. After identification of a neuron by brushing the face, graded mechanical stimuli were applied to RFs. To avoid sensitization by noxious stimulation, we did not use repeated noxious stimuli to search for high-threshold mechanosensitive neurons. Neuronal responses were saved on computer disk for subsequent off-line analysis of signals.

The waveforms of each neuron were amplified using a differential amplifier (AB-601G, Nihon Kohden, Tokyo, Japan, high cut: 10 KHz, low cut: 150 Hz) and identified using Spike 2 software (CED, Cambridge, UK). Peristimulus time histograms (bin width = 1 s) were generated in response to each stimulus. Background discharges were first recorded for 120 s before the application of the mechanical stimulation, and they were subtracted from the neuronal responses during the analysis. The mean firing frequency was calculated during mechanical stimulation. Afterdischarges were recorded for 10 s after pinching of the RF. The mechanical stimulation of the RFs was considered to have induced an effect when the mean firing frequency at 5 s after mechanical stimulation differed from mean background discharge rate by ± 2SD.

### Acute dissociation of TG neurons

The IAN-transected rats were tested for mechanical stimulation of the mental region at 14 days after nerve injury. Rats that showed allodynia-like responses to non-noxious mechanical stimulation of the mental skin were used for patch clamp recording experiments. For mental skin stimulation, mechanical stimulation was applied to adjacent regions more than 1 mm distant from the FG injection site.

Rats with mechano-allodynia were sacrificed by decapitation and TG neurons were used for the electrophysiological studies. Acute dissociation of TG neurons was performed as described previously [63,64]. Briefly, rats were anaesthetized with sodium pentobarbital (45 mg/kg, i.p.) and decapitated. The left TG was rapidly removed and incubated for 15-25 min at 37°C in modified Hank's balanced salt solution (130 mM NaCl, 5 mM KCl, 0.3 mM KH_2_PO_4_, 4 mM NaHCO_3_, 0.3 mM Na_2_HPO_4_, 5.6 mM glucose, 10 mM N-2-hydroxyethylpiperazine-N'-2-ethanesulfonic acid (HEPES), pH 7.3) containing collagenase type XI and type II (each 2 mg/ml; Sigma-Aldrich, MO, USA). The cells were dissociated by trituration with a fire-polished Pasteur pipette and then plated onto poly-L-lysine-coated coverslips in 35-mm dishes. The plating medium contained Leibovitz's L-15 solution (Invitrogen, Carlsbad, CA, USA) supplemented with 10% newborn calf serum, 26 mM NaHCO_3_, and 30 mM glucose. The cells were maintained in 5% CO_2 _at 37°C and used for recordings between 2 and 8 h after plating. After incubation, the coverslips were transferred to the recording chamber in a standard external solution containing 155 mM NaCl, 3 mM KCl, 1 mM CaCl_2_, 1 mM MgCl_2_, 10 mM HEPES and 20 mM glucose, pH 7.3.

### Recording solution and drugs

The composition of the extracellular recording solution used in these experiments is shown in Table 1. The cells were also studied in the presence and absence of TTX to determine which currents were TTX resistant. In these voltage-clamp experiments, 1 μM TTX was added to the extracellular solution, when TTX-R *I*_Na _was recorded in naive and IAN-transected rats. To examine the outward K^+ ^current, the solution was replaced with 150 mM choline chloride, 3 mM KCl, 1 mM MgCl_2_, 10 mM HEPES, and 20 mM glucose, pH 7.35 [24,63]. Some recordings were performed in the current-clamp mode and we used a quasiphysiological recording solution in this study (Table 1). In the current-clamp mode experiments, 1 μM TTX was added to the extracellular solution. All experiments were performed at room temperature (21-26°C).

**Table 1:** Composition of extracellular and intracellular solution.

*V*-clamp		*I*-clamp	
***Extracellular solution *(mM)**			
NaCl	30	NaCl	155
Choline chloride	50	HEPES	10
TEA	40	KCl	3
HEPES	10	CaCl_2_	1
MgCl_2_	3	MgCl_2_	1
Glucose	10	Glucose	20
			
Adjusted to pH = 7.4 with TEAOH		Adjusted to pH = 7.3 with NaOH	

			

***Intracellular solution *(mM)**			
CsF	110	Methane Sulfonic Acid	135
CsCl	40	KOH	130
TEA	40	KCl	20
HEPES	10	NaOH	15
NaOH	10	EGTA	2
EGTA	2	HEPES	7.5
MgCl_2_	2		
			
Adjusted to pH = 7.2 with CsOH		Adjusted to pH = 7.2 with KOH or HCl	

TEA: Tetraethylammonium chloride, EGTA: ethyleneglycol-bis (b-aminoethyl ether)-*N*, *N*, *N*', *N*',-tetra acetic acid.

### Patch-clamp recording

FG-labeled TG neurons were identified by applying a short pulse of UV light (340-380 nm) and capturing the image of fluorescent cells with a microscope (Nikon, Tokyo, Japan). Locally developed software permitted the superposition of a tracing of the perimeter of the fluorescent cell onto the image of the same cell in the TG visualized with visible light. Whole cell recordings were conducted with the rapid perforated-patch technique [24,50,67-69]. Fire-polished patch pipettes (2-5 MΩ) were filled with an internal solution (Table 1) and amphotericin B. In the case of the potassium current recording under voltage clamp, we used the same internal solution as described in current clamp condition (Table 1). Both current- and voltage-clamp recordings were conducted with an Axopatch 200B amplifier (Axon Instr., Foster City, CA, USA). Signals were low-pass filtered at 1 or 5 kHz and digitized at 10 kHz.

Neurons were always bathed in a flowing stream of external solution, except during the application of drugs. After seal formation and membrane perforation, leakage and capacitive transients were reduced by analog circuitry. A series resistance compensation (> 80%) was employed [70]. The recording chamber (volume, 0.5 ml) was mounted on an inverted microscope (Nikon, Tokyo, Japan) equipped with phase-contrast, a video camera and two micromanipulators. The chamber was perfused under gravity with an external solution at approximately 0.5 ml/min. Current density was determined by dividing the peak current evoked by the cell capacitance.

In the voltage-clamp mode, TTX-R *I*_Na _was recorded in naive and IAN-transected rats. TTX-S *I*_Na _was isolated by digitally subtracting TTX-R *I*_Na _from the total *I*_Na_. The current-voltage (*I-V*) relationship was first monitored by using step pulses (50 ms) from the holding potential of -80 mV to +80 mV in 5 mV increments at 5 s intervals.

In the current-clamp mode, we firstly determined the threshold (1T) for evoking action potential (overshoot of action potential > 30 mV). The threshold was defined as the current values for eliciting depolarizing single pulses (10-400 pA, 300 ms). The firing rate of action potentials was assessed by counting the number of action potentials evoked by the depolarizing pulses (1T, 2T and 3T). The spike amplitude and height of overshoot, and the threshold current, were also assessed in naive and IAN-transected rats as illustrated in Figure 5A.

We also analyzed the K^+ ^current in this model. We used voltage protocols modified from a previous study [24,63,64]. Outward K^+ ^currents were elicited by stepping to a conditioning voltage of either -40 mV or -120 mV from a holding potential of -60 mV; then the membrane was depolarized from -60 mV to +60 mV in increments of 10 mV; +60 mV produced the largest peak current in each recording. The transient A currents was determined by subtraction of -40 mV protocol from -120 mV protocol. Activation of the currents in standard solution was rapid and decayed only partially during 300 ms depolarization pulses. The amplitudes and rates of rise in the absolute current increased with increasing depolarization.

### Statistical analysis

Results are presented as median for behavioral test, and mean ± SEM for single fibers analysis, patch clamp analysis and FG immunohistochemistry. One-way ANOVA followed by Dunnett's test was used for data from the FG labeling of TG neurons. ANOVA on rank with post-hoc Student-Newman-Keuls test was used for the behavioral data. The Mann-Whitney U test, two-way ANOVA followed by the Dunn's and Holm-Sidak tests were used for data from the single fiber recording experiments, and Duncan's new multiple range test was used for the data from the patch clamp recording experiments. Differences were considered significant at *p *< 0.05.

## Competing interests

The authors declare that they have no competing interests.

## Authors' contributions

All authors read and approved the final manuscript. KN carried out the experiments and data analysis. MT, YT, MK, JK and MS helped the experiments, data analysis and paper writing. BJS and SM provided data interpretation and helped to finalize the manuscript. AK provided data interpretation. KI conceptualized the hypothesis, designed and supervised the experiments, directed the data analysis, and finalized the manuscript.
